# Accelerated Resolution of AA Amyloid in Heparanase Knockout Mice Is Associated with Matrix Metalloproteases

**DOI:** 10.1371/journal.pone.0039899

**Published:** 2012-07-10

**Authors:** Bo Wang, Ying-xia Tan, Juan Jia, Andreas Digre, Xiao Zhang, Israel Vlodavsky, Jin-ping Li

**Affiliations:** 1 Department of Medical Biochemistry and Microbiology, University of Uppsala, Uppsala, Sweden; 2 Department of Public Health and Caring Sciences, Molecular Geriatrics, University of Uppsala, Uppsala, Sweden; 3 Cancer and Vascular Biology Research Center, The Bruce Rappaport Faculty of Medicine, Technion, Haifa, Israel; Instituto Butantan, Brazil

## Abstract

AA-amyloidosis is a disease characterized by abnormal deposition of serum A amyloid (SAA) peptide along with other components in various organs. The disease is a complication of inflammatory conditions that cause persistent high levels of the acute phase reactant SAA in plasma. In experimental animal models, the deposited amyloid is resolved when the inflammation is stopped, suggesting that there is an efficient clearance mechanism for the amyloid. As heparan sulfate (HS) is one of the major components in the amyloid, its metabolism is expected to affect the pathology of AA amyloidosis. In this study, we investigated the effect of heparanase, a HS degradation enzyme, in resolution of the AA amyloid. The transgenic mice deficient in heparanase (Hpa-KO) produced a similar level of SAA in plasma as the wildtype control (Ctr) mice upon induction by injection of AEF (amyloid enhancing factor) and inflammatory stimuli. The induction resulted in formation of SAA amyloid 7-days post treatment in the spleen that displayed a comparable degree of amyloid load in both groups. The amyloid became significantly less in the Hpa-KO spleen than in the Ctr spleen 10-days post treatment, and was completely resolved in the Hpa-KO spleen on day 21 post induction, while a substantial amount was still detected in the Ctr spleen. The rapid clearance of the amyloid in the Hpa-KO mice can be ascribed to upregulated matrix metalloproteases (MMPs) that are believed to contribute to degradation of the protein components in the AA amyloid. The results indicate that both heparanase and MMPs play important parts in the pathological process of AA amyloidosis.

## Introduction

Amyloidosis is a heterogeneous group of diseases characterized by extracellular deposition of abnormal proteins in the form of fibrils. So far more than 20 proteins are identified to be able to form amyloid, including serum A amyloid protein (SAA). This apoliprotein is an acute phase reactant associated with high-density lipoprotein (HDL). In response to acute inflammation, the level of SAA in plasma is dramatically increased, often with 2–3 orders of magnitude (from 0.001 to 1 mg/ml plasma) [1]. Normally, the SAA level returns to the basal level after inflammation is stopped; however, a persist high plasma level of SAA in the circumstance of chronic inflammation may lead to aggregation of the protein, by a yet unknown mechanism, subsequently leading to abnormal deposition of the protein in organs such as the spleen, liver and kidney [2]. Thus, AA amyloidosis is a complication of chronic inflammatory diseases, e.g. rheumatoid arthritis and tuberculosis. The disease is rare, but often severe with high mortality due to functional failure of affected organs.

AA amyloidosis can be induced in mice by injection of amyloid enhancing factor (AEF) intravenously in combination with inflammatory stimulation, e.g. subcutaneous injection of silver nitrate [3]. This procedure can rapidly cause AA amyloid formation in the spleen and liver [4]. The amyloid is gradually resolved after the induction [5], indicating there is a spontaneous clearance mechanism.

Heparan sulfate (HS), a linear sulfated polysaccharide ubiquitously expressed on the cell surfaces and in the extracellular matrix, has been found as a prominent component in all amyloid deposits examined, e.g.SAA plaques, suggesting its participation in amyloidogenic pathology [6]. *In vitro* studies show that SAA aggregation is dependent on cellular HS [7], pointing to an active role for HS in SAA amyloidosis. Our early *in vivo* studies reveal that overexpressing heparanase, an endo-glucuronidase specifically cleaves HS and heparin, attenuated the mice to AA amyloidosis induced by injection of AEF [8], providing a direct evidence for a critical role of HS in SAA deposition. The hypothesis behind this finding is that the shorter fragments of HS produced in the liver of the Hpa-tg mice [9], due to extensive degradation by heparanase, failed to form complex with SAA, precluding SAA aggregation.

In this study, we assumed that the mice lacking heparanase (Hpa-KO) might produce more and persistent amyloid, because they produced non-degraded long HS chains due to lacking of heparanase cleavage [10]. Unexpectedly, the results show that the Hpa-KO and control (Ctr) mice produced comparable amount of amyloid upon the induction. Surprisingly, the amyloid was resolved more rapidly in the Hpa-KO than in the Ctr mice. Examination of the tissues revealed increased expression of matrix metalloproteases (MMPs) in the Hpa-KO mice. The data suggest a co-regulation between the proteases and heparanase, a mechanism likely responsible for maintaining the homeostasis of ECM. The result may propose a possible explanation to the clinical observation, that majority of the patients suffering from inflammation do not develop AA amyloidosis, owe to the effective clearance mechanism. Thus, it should be of interest in clinic to examine the patients who developed AA amyloidosis for the expression of MMPs and heparanase.

## Results

### Mice Deficient in Heparanase Exhibited Rapid Resolution of SAA Amyloid

**Figure 1:**
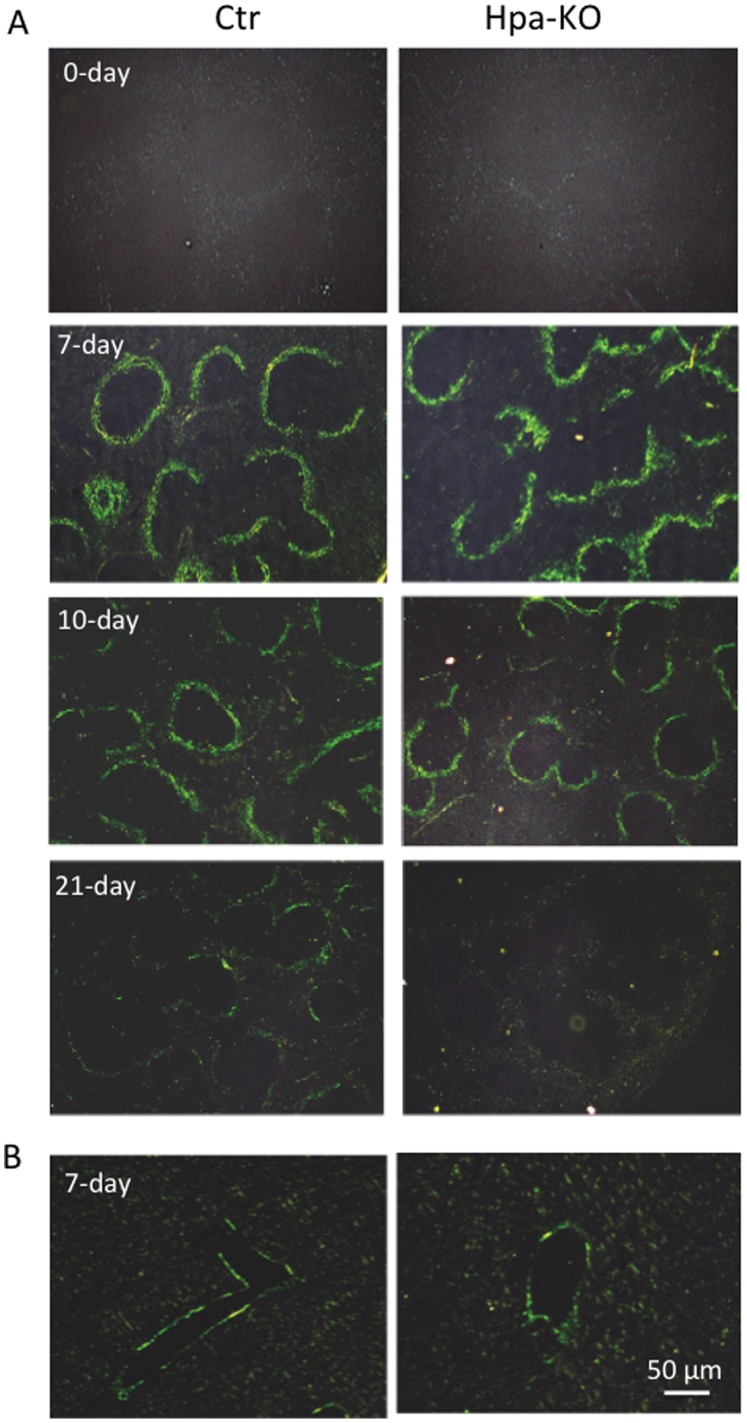
Congo red staining of tissue sections. Hpa-KO and C57bl (Ctr) were treated by injection of AEF (amyloid enhancing factor) and silver nitrate (to stimulate inflammation). Following the treatment, the animals were sacrificed on the days indicated each panel. Paraffin-embedded sections (5 µm) from spleen (A) and liver (B) were stained with Congo red and visualized under polarized light. Spleen sections from non-treated animals are shown in the upper panels. Original amplification 200x.

To induce AA amyloidosis, mice, C57bl/6 (Ctr) and heparanase-knockout (Hpa-KO), at the age of 12 weeks were injected with 100 µg of amyloid enhancing factor (AEF) into the tail vein, followed by subcutaneous injection of 0.2 ml of 1% silver nitrate to stimulate inflammation. Seven days later, the liver, kidney and spleen were collected for examination of amyloid formation. Congo red staining revealed that all animals treated with AEF developed substantial amyloid in the spleen, to a similar degree for both groups (Figure 1A); in comparison, moderate amyloid formation was detected in the liver (Figure 1B), and essentially no amyloid in the kidney of both groups (Figure S1). The experiment was repeated with 20 mice in each group and 4–5 animals from each group were sacrificed on 10, 21, 42 and 72 days post treatment for examination of amyloid resolution. The spleen collected on days 42 and 72 post-treatment did not show any Congo red positive signals in both groups, indicating a complete clearance of the amyloid from the organs. Notably, on day 21 post-treatment substantial Congo red positive signals were detected in the Ctr spleen; in contrast, the Hpa-KO spleen showed virtually no amyloid (Figure 1A). This difference is also observed in the tissues collected on day 10, where the Congo red signals are markedly stronger in the splenic follicle of Ctr than in that of the Hpa-KO (Figure 1A).

**Figure 2:**
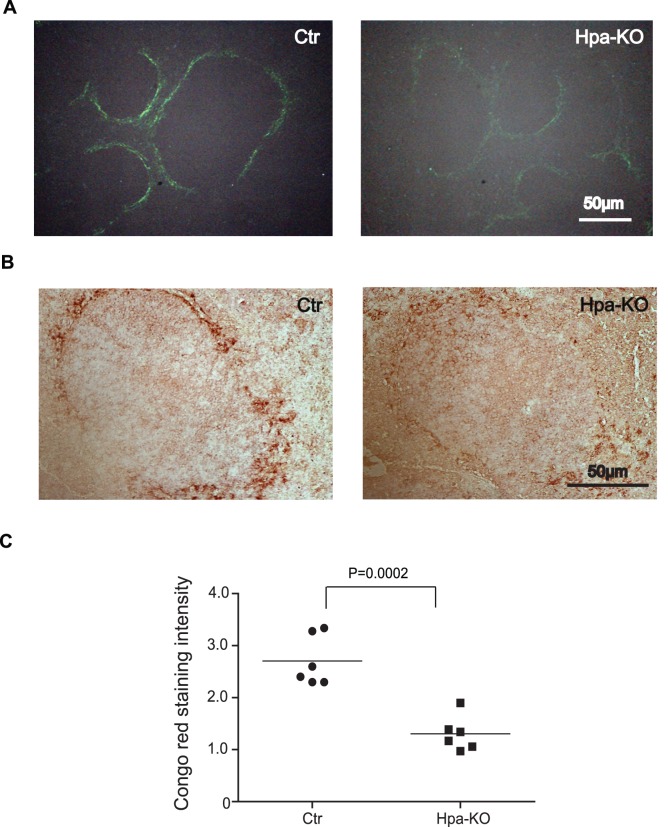
Quantification of SAA amyloid in the spleen. The mice (n = 6 in each group) were sacrificed on day 14 post-induction. The sections of the spleen (5 µm) were analyzed by Congo red staining (A) and immunohistostaining with anti-SAA antibody (B). Original amplification 400x. The intensity of Congo red staining was quantified under the fluorescence microscopy with Image J (C).

To determine the amyloid load in the spleen, additional 6 Ctr and 6 Hpa-KO were induced. Examination of the spleens collected on day14 after induction reveals, again, a substantial difference in amyloid load between the two groups. The representative sections of the spleen from these mice show appreciably stronger signals of Congo red staining in the Ctr spleen than in the Hpa-KO spleen (Figure 2A). The deposition of SAA in the Congo red positive spleen was confirmed by immunohistostaining with an anti SAA antibody, showing coherent results of stronger signals in Ctr than in Hpa-KO spleen (Figure 2B). Notably, a difference is observed that the Congo red stained signals are preferably in the perifollicular areas of the spleen, while the SAA immunohistostaining signals are more diffused, particular in the Hpa-KO spleen. This may indicate a resolving process where the degraded non-fibril SAA was only detected by the anti-SAA antibody, not by Congo red staining. Quantification of the Congo red intensity in the sections as described in the Materials and Methods confirms the significant less amyloid load in the Hpa-KO than in the Ctr spleens (Figure 2C).This is confirmed by Western blot analysis of the insoluble aggregates in the tissue lysate. The insoluble amyloid was extracted by incubation of the pellet in 5% SDS at 60°C overnight, and the resultant extracts were subjected to Western blot analysis. Immunoblotting with the anti-SAA antibody revealed a migration smear of the samples, indicating the nature of AA with different aggregation degree (Figure S2A). The samples of Ctr produced, in spite of a variation, overall stronger signals than that of Hpa-KO, demonstrating higher level of aggregate in the Ctr tissue. Interestingly, comparable amount of SAA was detected in the soluble fractions of the tissue lysate (Figure S2C), suggesting that the degraded amyloid is rapidly metabolized in both groups.

### Matrix Metallproteases Contribute to the Accelerated Amyloid Resolution in Hpa-KO Mice

**Figure 3:**
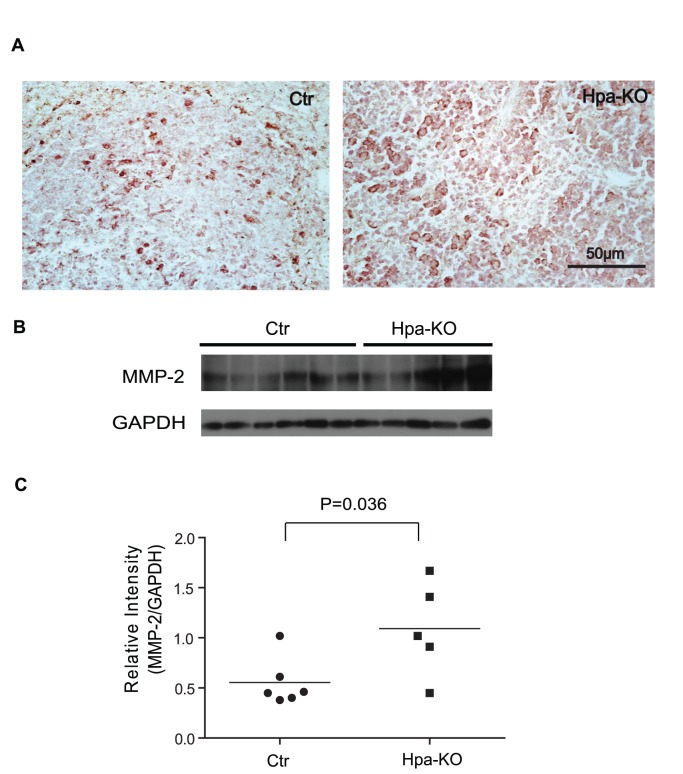
Detection of MMP-2 in the spleen. The sections from the spleen collected 14-days post-treatment were immunostained with anti MMP-2 antibodies (A). Original amplification 400x. The frozen tissues of the corresponding organ were analyzed by Western blot using the same antibodies (B). The band intensity from (B) was quantified with ImageJ and expressed as the ratio with GAPDH (C).

To find out the mechanism for the difference of SAA resolution in Hpa-KO and Ctr groups, the sections of the spleens were analyzed for expression of matrix metalloproteases (MMPs). Immunohistostaining detected stronger MMP-2 expression in the Hpa-KO spleen in comparison to that in the Ctr spleen (Figure 3A). Western blot quantification confirms a significant higher level of MMP-2 in the Hpa-KO spleen than in the Ctr spleen (Figure 3B,C). MMP-9 was expressed at the similar level in the spleen of both groups (Figure S3).

**Figure 4:**
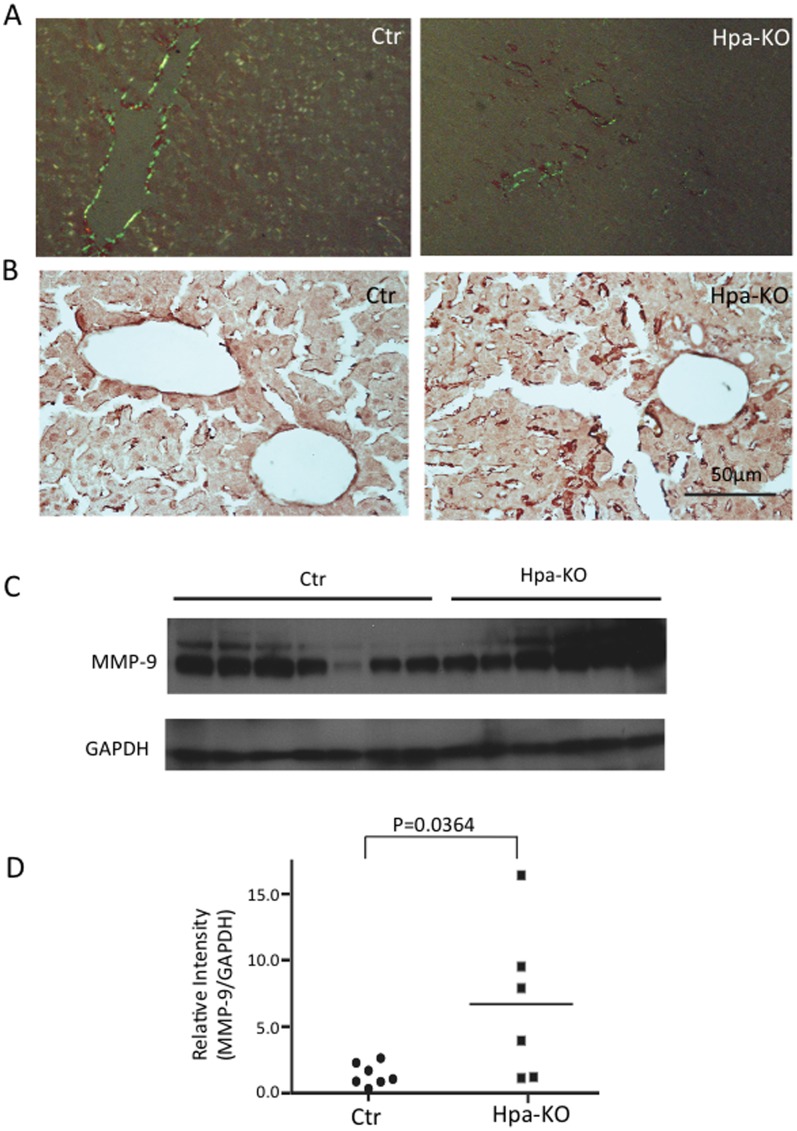
Amyloid deposition and MMP expression in the liver. The sections from the liver collected 14 days post treatment were stained with Congo red and visualized under polarized light (A). The adjacent sections were immunostained with anti MMP-9 antibodies (B). Original amplification 400x. The frozen tissues of the corresponding organ were analyzed by Western blot using the same antibodies (C). The band intensity from (C) was quantified by the ImageJ software and expressed as the ratio with GAPDH (D).

Examination of the liver collected on day 14 post-induction revealed, again, less amyloid in the Hpa-KO groups (Figure 4A). Analysis of MMPs by immunohistostaining found, in contrast to the spleen, that MMP-2 was expressed to a similar pattern in both Hpa-KO and Ctr livers (Figure S4). Conversely, MMP-9 immunostaining resulted in a much stronger signal in the Hpa-KO liver (Figure 4B), which is confirmed by Western blot quantification (Figure 4C,D). Q-PCR analysis corroborated the finding for upregulated expression of MMP-9 in the amyloid-load liver (Figure S5).

**Figure 5:**
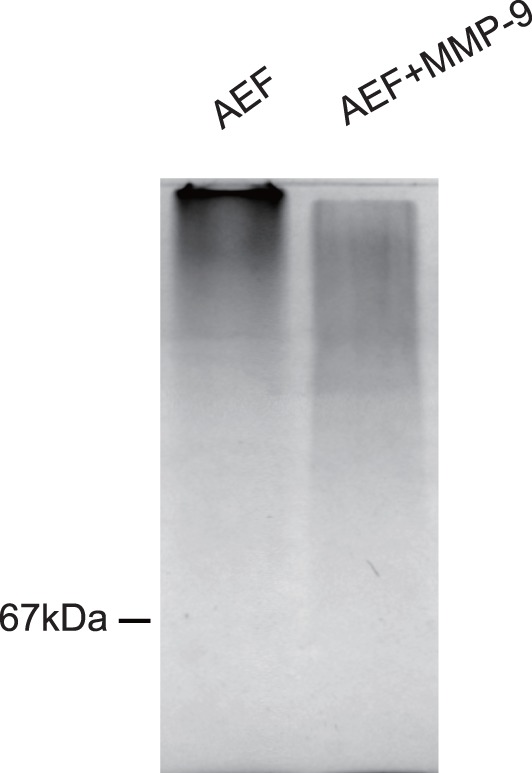
MMP-9 degradation of AEF. Amyloid enhancing factor (AEF) isolated from the AA amyloid-laden spleen was incubated with and without presence of MMP-9, and analyzed by SDS-PAGE (8%) that is stained with Coomassie blue. Migration corresponding to bovine serum albumin (67 kDa) was indicated.

To demonstrate that MMP indeed degrades amyloid, AEF prepared from mouse spleen (the same preparation as used for induction) was incubated with MMP-9 (Enzo Life Sciences, USA). SDS-PAGE analysis of the incubated samples clearly shows a degradation of the AEF by MMP-9 (Figure 5).

### SAA Plasma Level is not Affected by Heparanase Expression

**Figure 6:**
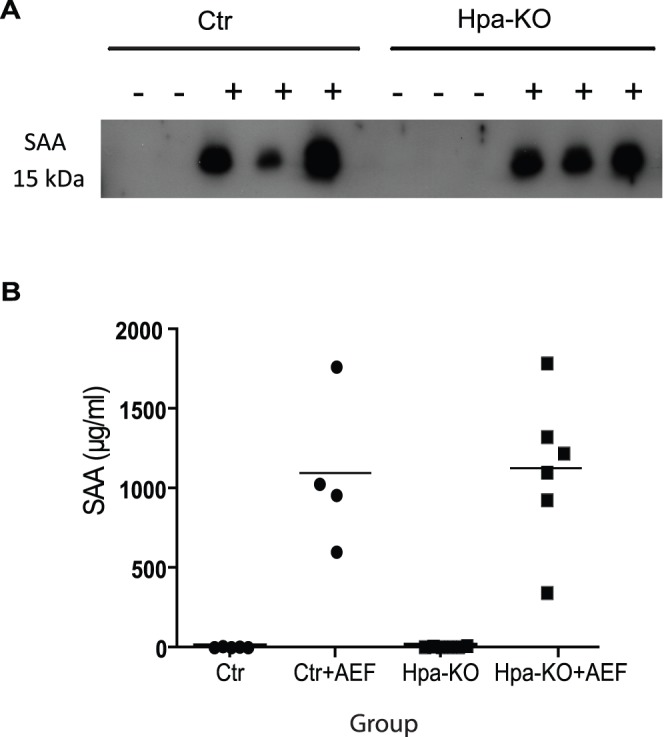
Analysis of SAA in blood plasma. (A) Blood was collected from mice by heart punctuation into citrate-anticoagulation tubes on day 3 post-treatment. After removal of blood cells by centrifugation, the plasma (1 µl) was separated on a 8% SDS-PAGE and probed with the anti-SAA antibody (see Materials and Methods). (B) Quantification of SAA in the plasma with the Mouse SAA ELISA Kit. The standard curve was performed with the reagents included in the kit.

To find out whether lacking of heparanase has an impact on SAA production in response to inflammation, we have determined the SAA levels in the plasma from mice before and 3 days after the induction. Western blot analysis using the anti-SAA antibody detected no SAA in the plasma of any mouse before induction, but strong signals in the plasma of both groups after the induction (Figure 6A). Quantification with the Mouse SAA ELISA Kit shows that the mice before induction had essentially non-detectable SAA in the plasma (Figure 6B), in accordance with the Western blot analysis. Upon induction, the level of SAA is dramatically increased, reaching a concentration about 1 mg/ml. There is no difference between the two groups.

## Discussion

Accumulated information ratifies that heparan sulfate (HS) is essential for AA amyloidosis [8], [10]. As heparanase modulates, by degradation, the structure and, accordingly, the functions of HS [9], it is anticipated that elimination of heparanase may have an impact on AA amyloidosis. In this study, we assumed that the non-degraded long HS chains in heparanase deficient mice (Hpa-KO) might promote AA amyloidosis. Unexpectedly, the heparanase Hpa-KO and the control animals (Ctr) displayed a non-distinguishable spatial and temporal pattern in development of AA amyloidosis upon induction by injection of AEF and silver nitrate. No substantial amyloid was detected in any of the mice 3-days after the treatment, and comparable amount amyloid was found, primarily, in the spleen of all animals 7-days post-treatment. Together with our earlier findings [8], this result implies that the activity of HS in participating AA aggregation and deposition requires a minimal chain length, but is not proportional to the chain length. The HS in normal animals have a sufficient chain length to induce AA amyloidosis, a function that cannot be promoted by increase in length of the polysaccharide chain, as the case in the Hpa-KO mice. This is agreed with the observation by *in vitro* experiment where heparin fragments shorter than 12-mers failed to induce SAA to aggregate, while the fragments longer than 18-mers did not show difference from full-length heparin (50-sugar units) in promoting SAA aggregation (unpublished data).

The amyloid formed in the spleen, both in the Hpa-KO and Ctr animals, is completely resolved 42-days after the induction. This is in accordance with previous reported observation [5], that animals resolve the amyloid when inflammation ceases. Notably, we found that this resolution process is accelerated in the Hpa-KO mice, as the amyloid load was less in the spleen as well as the liver of Hpa-KO mice during 10–21 days post treatment (Figure 1). Quantification of the Congo Red positive signals in the spleen sections reveal a significant lower AA amyloid in the Hpa-KO spleen on day 14 after treatment (Figure 2C). The low AA amyloid level in the Hpa-KO spleen was verified by immunohistostaining with anti-SAA antibody and Western blot analysis of the tissue lysate. This result is in contradiction to our initial hypothesis that the longer HS chains in Hpa-KO mice would lead to persistent amyloidosis. This unexpected result led us to look for the factors that promoted resolution in Hpa-KO mice.

Amyloidosis is, primarily, caused by abnormal aggregation of the SAA protein produced during inflammation, along with other molecules including extracellular proteins and HS proteoglycans. Matrix metalloprotesases (MMPs), the major protease family in the extracellular matrix, are detected in the AA amyloid deposits [11], and it was reported that SAA was able to induce production of MMPs [12], [13]. Since increased MMP expression was also observed in transthyretin amyloidosis [14], suggesting that expression of MMPs maybe a self-regulation mechanism for degradation of amyloid. Our results by immunochemical staining and Q-PCR clearly show an increased expression of the MMPs in the amyloid organs, providing *in vivo* evidence for such a regulation. The differential upregulation of MMPs in organs is of worth noting, suggesting that the regulatory mechanisms may be tissue specific. Our early study found elevated expression of MMP-2 and -9 in the liver of Hpa-KO mice [10], which is confirmed in this study (Figure S5). MMPs is known to play important roles for modulation of extracellular matrix structure and participate in pathological process such as cancer and arthritis [15]; putting together, this study further implies an interplay of heparanase with MMP, which affects AA amyloid resolution.

AA amyloidosis is a complication of inflammation that is a common condition associated with different types of diseases. Fortunately only a minor population of the patients suffering from inflammation develops AA amyloidosis. Animal experiments show that AA protein aggregation is unlikely to be spontaneous, but requires a ‘seed’, e.g. an aggregated amyloid protein, to initiate the aggregation. Therefore AEF is needed, in addition to inflammatory stimulation, to induce AA amyloidosis in mouse. However, an alternative scenario can occur in clinic, that patients under inflammatory conditions may develop amyloidosis; nevertheless, the amyloid can be removed effectively in most individuals when the inflammation ceases. The patients who developed into irreversible amyloidosis maybe a result of multiple factors including defect in the process of resolution.

Comparing to the common amylododic diseases, e.g, Alzheimer’s disease and type II diabetes, AA amyloidosis is a relatively rare, yet incurable disease. In many individuals with AA amyloidosis, adequate suppression of SAA production is not possible; thus there is a need for other therapies targeting aggregation and resolution. Our results show a clear resolving process of AA amyloid in mice, where MMPs play an active role. It is proposed that the level of MMPs should be monitored for the patients with chronic inflammation such as rheumatoid arthritis, which may serve as a marker for a potential risk of AA amyloidosis.

## Materials and Methods

### Animals

The homozygous heparanase knockout (Hpa-KO) mice were generated as described [10] and backcrossed to C57Bl/6 for more than 10 generations. The mice, C57Bl/6 and Hpa-KO, were maintained in the animal facility at the Biomedical Center, Uppsala. The animals were 10 to12-weeks-old at the induction point. The experiments were conducted in accordance with local ethic regulations for animal welfare (approval number C42/9).

### Induction of Serum A amyloidosis

AEF (amyloid enhancing factor) was prepared from the spleen of AA amyloidosis as previously described [16]. For induction, the animals were intravenously injected with 0.1 ml AEF (100 µg) in the tail vein or intraperitoneal followed by a subcutaneous injection of 0.2 ml 1% silver nitrate. The animals were sacrificed on days as described in respective figures. Part of the spleen and liver tissues were fixed in 4% formaldehyde for preparing tissue sections and the rest tissues were rapidly frozen in dry ice for preparation of tissue extract and RNA.

### ELISA Analysis

Blood was collected into 10% sodium citrate anticoagulant tubes by heart puncture and centrifuged to remove blood cells. SAA was measured using the Mouse SAA ELISA Kit (Tridelta Development Ltd., Co. Kildare, Ireland) according to manufacturer’s instruction.

### Immunohistostaining and Histochemistry

Paraffin-sections were deparaffinized and rehydrated at 10-minute intervals through alcohol baths of decreasing concentration (99.9%–70%). Immunostaining with antibodies against SAA (a gift from Dr. G. Westermark, Uppsala University), MMP-9 (Abcam, UK) and MMP-2 (Santa Cruz, USA) were performed on 5-µm tissue sections. Antigens were retrieved in 25 mM citrate buffer (pH7.2) by microwaving. Sections were blocked with Rodent Block M (Biocare Medical). Primary antibody incubation was carried out overnight at 4°C followed by incubation with secondary antibody for 60 min at room temperature. NOVA™ red reagents (Vector Labs) were used to visualize the immune signals. Congo red staining was performed as described [8] and the intensity of the staining was quantified by ImageJ under fluorescence microscope.

### Western Blot Analysis

The frozen tissues were homogenized in 1∶10 (w/v) CelLytic™ MT Cell Lysis Reagent with protease inhibitor cocktail (Sigma-Aldrich) followed by centrifugation. The insoluble pellet was further incubated in 50 µl of 5% SDS at 60°C overnight. The extract was subjected to SDS-PAGE (8% or 12%) and Western blotting. For analysis of blood plasma, 1 µl of the plasma (see above) was use for Western blot. For all Western blot analysis, the samples were blot onto a membrane that is blocked in 5% nonfat dry milk. Then the membranes were probed with primary antibodies followed by the corresponding secondary antibodies. Signals were visualized using SuperSignal West Pico or Dura substrates (Thermo). Quantitative band analysis was performed with ImageJ software.

### Real-time Quantitative Analysis of Gene Expression

The total RNA was prepared from the frozen liver collected from mice of non-induced and 14-days post-induction with the E.Z.N.A total rNA kit I (OMEGA bio-tek). The cDNA prepared using the IScript cDNA Synthesis kit (Bio-Rad) was used for qPCR (SSoFast EvaGreen Supermix, Bio-Rad) quantification with the primers described in [10]. The data were normalized using the mRNA level of GAPDH from each sample.

### In vitro MMP-9 Degradation of AEF

AEF (12 µg) was incubated with or without MMP-9 (0.9 µM) at 37°C for 3 hr. The incubations was mixed with SDS sample buffer and denatured at 100°C for 5 min. The samples were separated by SDS-PAGE (8%) followed by Coomassie blue staining.

### Statistical Method

Two-tailed unpaired Student’s t test was used to determine the significance between population means. Statistical significance was set at *P*<0.05.

## Supporting Information

Figure S1 **Congo red staining of kidney sections.** The kidney sections from Hpa-KO and C57bl (Ctr) mice 7-days after induction were stained with Congo red. Original amplification 200x.
(TIFF)

Figure S2 **Western blot analysis of soluble and insoluble SAA in the spleen.** The frozen half of the spleens were lysed and centrifuged. After collection of the supernatant, the pellet was incubated in 5% SDS buffer for extraction of the insoluble aggregates. Both the insoluble (A) and soluble (C) fractions were analyzed by Western blot. Average band intensity of the 4 samples analyzed in (A) and (C) are shown in B and D, respectively. The quantification was done with ImageJ. Actin was not detected in the insoluble fractions; 15 µg total protein in each sample is applied.
(PDF)

Figure S3 **Detection of MMP9 in the spleen**. The sections from the spleen collected on14-days post-treatment (adjacent sections as in Fig. 3) were immunostained with anti MMP-9 antibodies (A). Original amplification 400x. The frozen tissues of the corresponding organs were analyzed by Western blot using the same antibodies (B). The band intensity from (B) was quantified with ImageJ and expressed as the ratio with GAPDH.
(PDF)

Figure S4 **Detection of MMP2 in the liver.** The sections from the liver collected on day 14 post-treatement (same as in Fig. 4) were stained with anti MMP-2 antibodies (A). Original amplification 400x. The frozen tissues of the corresponding organs were analyzed by Western blot using the same antibodies (B). The band intensity from (B) was quantified with ImageJ and expressed as the ratio with GAPDH.
(PDF)

Figure S5 **Q-PCR analysis of MMP expression.** Total RNA was extracted from the liver of normal and induced mice (the same tissue as used for Figure 4), and subjected to quantitative real time PCR analysis. The relative expression level of the genes (in reference to GAPDH) in the Ctr or non-treated animals was regarded as 1.
(PDF)
