# Supplementary figures and images for: Accelerated Resolution of AA Amyloid in Heparanase Knockout Mice Is Associated with Matrix Metalloproteases
Source: PLoS One. 2012 Jul 10;7(7):e39899. doi: 10.1371/journal.pone.0039899 (PMC3393718; doi:10.1371/journal.pone.0039899)

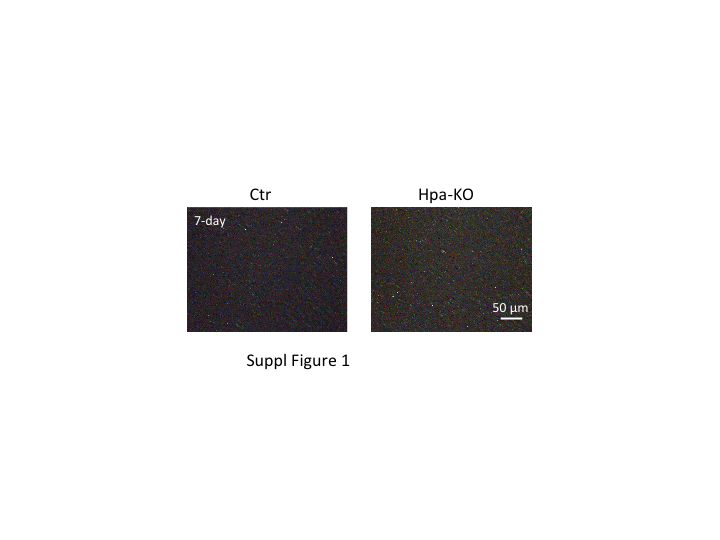

Supplement: Figure S1 — Congo red staining of kidney sections. The kidney sections from Hpa-KO and C57bl (Ctr) mice 7-days after induction were stained with Congo red. Original amplification 200x. (TIFF) [file pone.0039899.s001.tif]

A

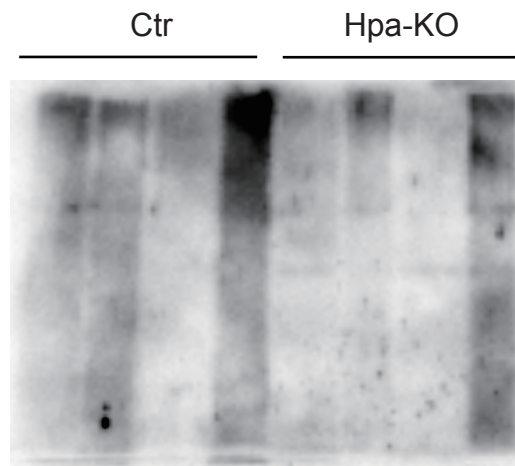

B

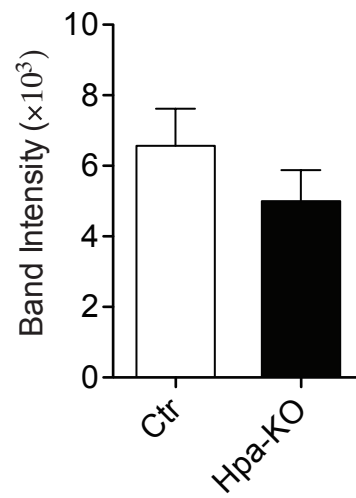

C

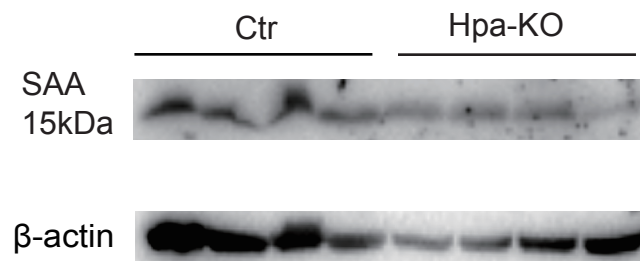

D

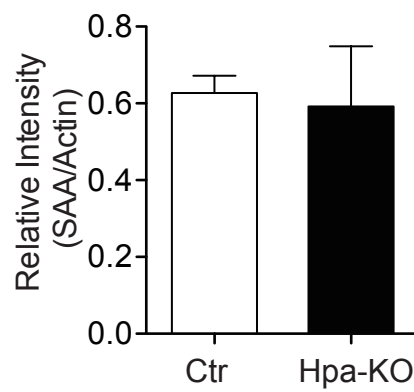

Suppl Figure 2

Supplement: Figure S2 — Western blot analysis of soluble and insoluble SAA in the spleen. The frozen half of the spleens were lysed and centrifuged. After collection of the supernatant, the pellet was incubated in 5% SDS buffer for extraction of the insoluble aggregates. Both the insoluble (A) and soluble (C) fractions were analyzed by Western blot. Average band intensity of the 4 samples analyzed in (A) and (C) are shown in B and D, respectively. The quantification was done with ImageJ. Actin was not detected in the insoluble fractions; 15 µg total protein in each sample is applied. (PDF) [file pone.0039899.s002.pdf]

**A**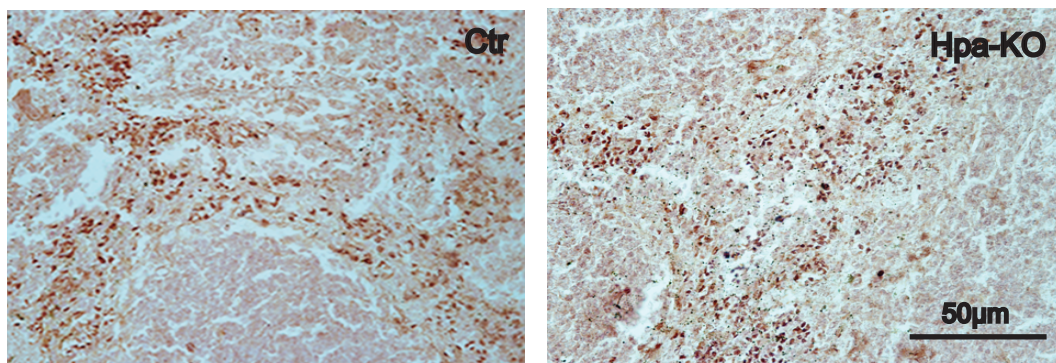**B**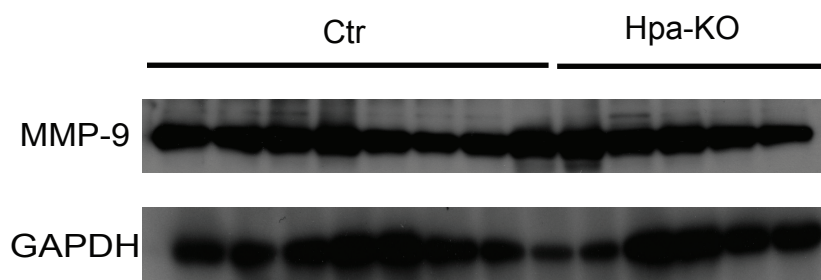**C**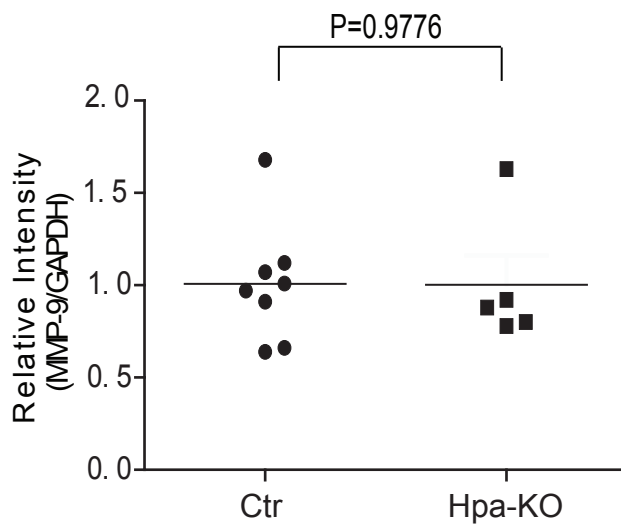

Suppl Figure 3

Supplement: Figure S3 — Detection of MMP9 in the spleen. The sections from the spleen collected on14-days post-treatment (adjacent sections as in Fig. 3) were immunostained with anti MMP-9 antibodies (A). Original amplification 400x. The frozen tissues of the corresponding organs were analyzed by Western blot using the same antibodies (B). The band intensity from (B) was quantified with ImageJ and expressed as the ratio with GAPDH. (PDF) [file pone.0039899.s003.pdf]

**A**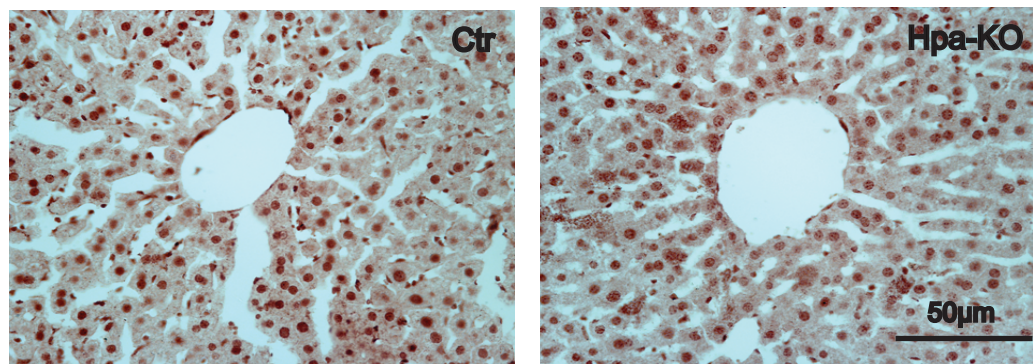**B**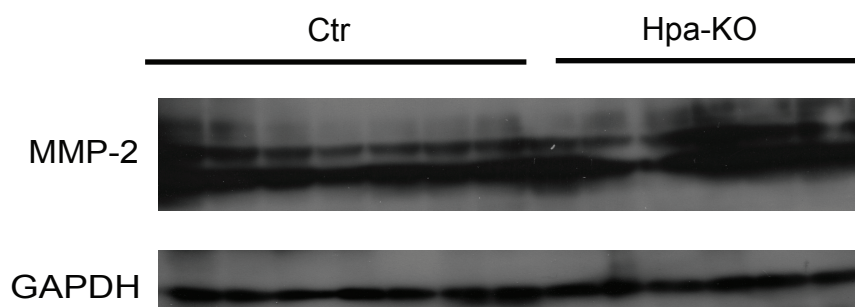**C**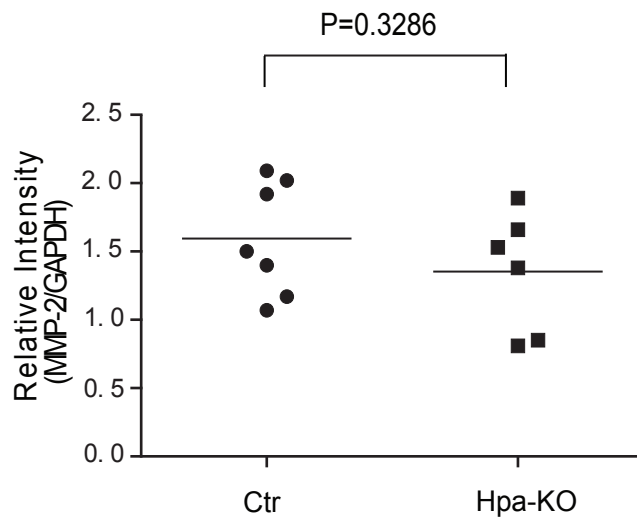

Suppl Figure 4

Supplement: Figure S4 — Detection of MMP2 in the liver. The sections from the liver collected on day 14 post-treatement (same as in Fig. 4) were stained with anti MMP-2 antibodies (A). Original amplification 400x. The frozen tissues of the corresponding organs were analyzed by Western blot using the same antibodies (B). The band intensity from (B) was quantified with ImageJ and expressed as the ratio with GAPDH. (PDF) [file pone.0039899.s004.pdf]

A

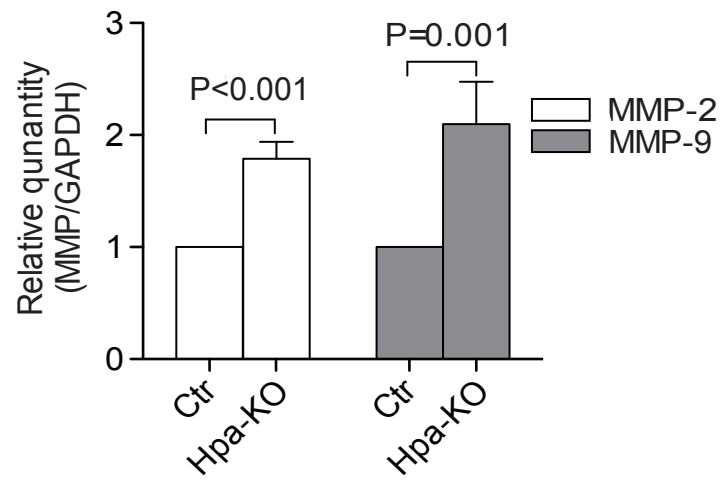

B

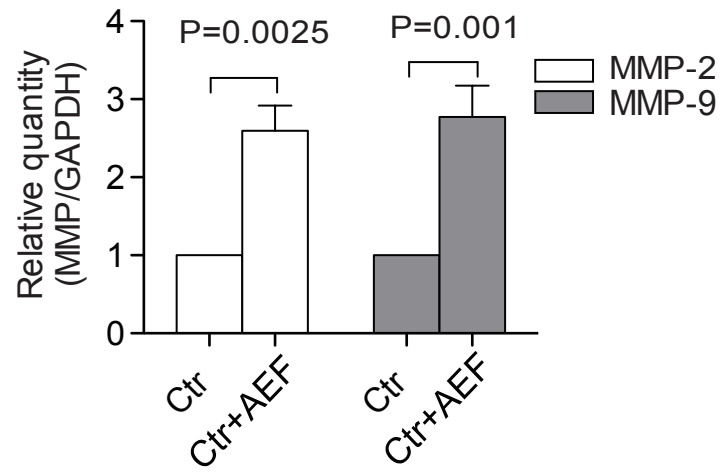

C

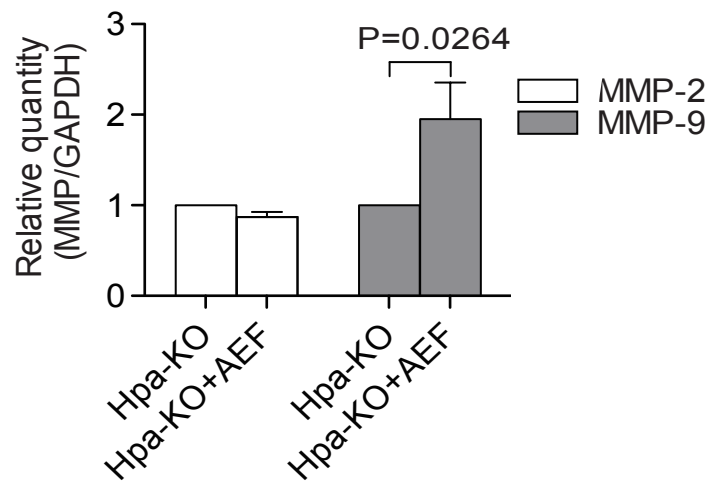

Suppl Figure 5

Supplement: Figure S5 — Q-PCR analysis of MMP expression. Total RNA was extracted from the liver of normal and induced mice (the same tissue as used for Figure 4), and subjected to quantitative real time PCR analysis. The relative expression level of the genes (in reference to GAPDH) in the Ctr or non-treated animals was regarded as 1. (PDF) [file pone.0039899.s005.pdf]
